# The addition of neoadjuvant pertuzumab for the treatment of HER2+ breast cancer: a cost estimate with real-world data

**DOI:** 10.1186/s13561-021-00332-0

**Published:** 2021-09-10

**Authors:** Andreia Borges, Filipa Pereira, Patrícia Redondo, Luís Antunes, Cláudia Vieira, Pedro Antunes, Maria José Bento, Susana Sousa, José Machado Lopes, Francisco Rocha-Gonçalves, Joaquim Abreu de Sousa, Deolinda Sousa Pereira, Marina Borges

**Affiliations:** 1 Outcomes Research Lab, Portuguese Oncology Institute of Porto (IPO Porto), Porto, Portugal; 2 Management, Outcomes Research, and Economics in Healthcare Group, IPO Porto Research Center (CI-IPOP), Portuguese Oncology Institute of Porto (IPO Porto), Porto, Portugal; 3 Medical Oncology Department, Portuguese Oncology Institute of Porto (IPO Porto), Porto, Portugal; 4 Department of Epidemiology, Portuguese Oncology Institute of Porto (IPO Porto), Porto, Portugal; 5 Cancer Epidemiology Group, IPO Porto Research Center (CI-IPOP), Portuguese Oncology Institute of Porto (IPO Porto), Porto, Portugal; 6 Molecular Oncology Group, IPO Porto Research Center (CI-IPOP), Portuguese Oncology Institute of Porto (IPO Porto), Porto, Portugal; 7 Faculty of Medicine, University of Porto, Porto, Portugal; 8 Surgical Oncology Department, Portuguese Oncology Institute of Porto (IPO Porto), Porto, Portugal; 9 Department of Population Studies, Institute of Biomedical Sciences Abel Salazar, University of Porto, Porto, Portugal; 10 Luz Saúde, Porto, Portugal

**Keywords:** Pertuzumab, Neoadjuvant treatment, HER2-positive breast cancer, Cost-effectiveness analysis

## Abstract

**Background:**

Breast cancer (BC) is largely prevalent worldwide. HER2-positive BC account for roughly 20–25% of all BC cases and has an overall survival lower than other BC. Innovation on BC therapeutics is a constant, but novel therapies have higher costs. Therefore, cost-effectiveness research is essential to provide healthcare decision-makers with solid foundations for a resource allocation. This study aims to estimate the average direct medical costs/patient and cost-effectiveness of adding pertuzumab in neoadjuvant treatment (NeoT) for HER2-positive breast cancer (BC).

**Methods:**

Two retrospective real-world consecutive cohorts of ≥18yo female patients diagnosed with HER2-positive BC treated with NeoT at the Breast Clinic of IPO-Porto were studied. The AC-DH regimen (2012–2015) comprised 8 cycles of neoadjuvant therapy (4 cycles of doxorubicin + cyclosphosphamide followed by 4 cycles ofdocetaxel + trastuzumab), while the AC-DHP regimen (2015–2017) included also pertuzumab as NeoT. NeoT was followed by surgery and adjuvant trastuzumab. Micro-costing technique and a bottom-up approach was used comprising all medical direct costs from the hospital perspective. Unit costs were obtained from government official prices or from IPO-Porto costing system. Costs were adjusted to 2017 and are expressed in euros. Multivariable logistic regression models were used for effectiveness assessment, while generalized linear models with gamma distribution were used for costs. ICER was calculated using the pathological complete response (pCR) as the preferential measure of effectiveness. Sensitivity analysis was also performed.

**Results:**

AC-DHP (*n* = 40) and AC-DH (*n* = 54) cohorts had heterogenous patient profiles (median age 43y/53y; 67.5%/59.3% positive HR; 60.0%/27.8% operable; 25.0%/24.1% inflammatory, respectively). The AC-DHP average total cost/patient was 56,375€, with pertuzumab accounting for 13,978€ (24.79%) and increasing in 15,982€ the average cost/patient (*p* < 0.001). Clinical staging and hormone receptors (HR) were significantly associated with pCR. ICER was 1.370€ per percentage point of pCR.

**Conclusions:**

ICER was more favourable in stage III HR negative BC patients compared to other patient profiles. Innovative treatments access is critical to deliver high-quality healthcare, but sustainability must be considered. These results suggest the importance of establishing a cost-effectiveness profile of Pertuzumab in NeoT for HER2-positive BC.

**Supplementary Information:**

The online version contains supplementary material available at 10.1186/s13561-021-00332-0.

## Highlights

i. The results obtained in the AC-DHP cohort, which included Pertuzumab as a NeoT, suggest better clinical outcomes compared to the AC-DH group, a confirmation based on real-world data.
ii. Some patient profiles seem to be more promising concerning the overall costs for pathological complete response, but the present data does not include a long-term follow-up period, which suggests that interpretations should be performed with caution.
iii. Pertuzumab cost estimated represented a great proportion of overall total costs per patient, but further research (including the costs of the whole episode of care) and continuously monitoring (additional RWE) is needed for a more robust cost-effectiveness analysis.

**Supplementary Information:**

The online version contains supplementary material available at 10.1186/s13561-021-00332-0.

## Background

Breast cancer (BC) is the most common cancer in women in the world. It affects more than 2 million women globally each year and represents approximately 15% of female cancer deaths [1]. In Portugal, BC was responsible, in 2018, for 12% of all cancer cases and was the leading cause of cancer in women, reaching an incidence of 128.7/100,000 [2]. Death rate data from 2017 puts Portugal (34.4 deaths per 100,000) behind its neighbour Spain (28.5 deaths per 100,000), but ahead of other wealthy nations like France, Germany and the United Kingdom [3]. However, the disease was still responsible for 3.4% (3.2–3.6%) of all female deaths in Portugal in 2017 [3].

Like in other cancers, the histological subtypes in BC impact in the therapeutic approach and in the expected clinical outcomes. The overexpression of the human epidermal growth factor receptor (HER) 2 protein, commonly known as HER2-positive BC, is present in approximately 20–25% of BC cases and is associated with worse prognosis and, consequently, overall survival (OS) [4].

The treatment success in HER2-positive BC was improved with the use of trastuzumab as neoadjuvant and adjuvant therapy. In fact, the introduction of trastuzumab has transformed the natural history of the disease for these patients [5]. Nonetheless, still approximately 25–30% of HER2-positiveBC treated patients still experience relapse during follow-up after adjuvant therapy with trastuzumab [5, 6]. In this sense, new therapeutic options, such as pertuzumab, have been developed [7].

Pertuzumab is an anti-HER2 monoclonal antibody initially approved for the treatment of patients with HER2-positive metastatic or locally recurrent unresectable BC. Later, in 2015, the indication was extended and comprised the neoadjuvant treatment (NeoT) of adult patients with HER2-positive BC, locally advanced, inflammatory, or early stage BC at high risk of recurrence [8]. A second extension of the indication was approved in 2018 for the adjuvant treatment of early BC at high risk of recurrence and metastatic BC [9].

Neoadjuvant pertuzumab has shown positive efficacy and safety results [10, 11], and promising progression-free survival and disease-free survival after 5 years [12]. However, in terms of health economic data, available literature is mainly based on economic models populated with clinical trial data and estimations of resource utilization [13–18], demonstrating the necessity of generating real-world data (RWD) on pertuzumab utilization. In Portugal, the local authority INFARMED has not yet published a Health Technology Assessment (HTA) appraisal of pertuzumab for the NeoT of HER2-positive BC in early stages. Nonetheless, patients might have access to treatment under special authorization and case by case review by INFARMED.

Considering the scenario of increasing health expenses and resources constraints in National Health System (NHS), a reference public large-sized comprehensive cancer center plays an important role in monitoring cancer treatments effectiveness and in estimating its costs, so assessing the impact of a new technology is necessary not only for hospital budgets, but for HTA bodies. The assessment of the drug performance according to an institution’s clinical practice is, therefore, important for assessing the real benefit of a newly implemented technology, so that new payment models based on its outcomes can be implemented, in a value-based healthcare (VBHC) perspective. Hence, patients can benefit from new technology while assuring the sustainability of the NHS, which is currently a major concern.

To our best knowledge, the cost per patient of pertuzumab in neoadjuvant context was not yet calculated using real-world patient level data, thus this research aims to estimate the average direct medical costs per patient by adding pertuzumab in the clinical practice to the NeoT of HER2-positive BC patients, as well as to calculate the weight of pertuzumab in the overall costs. Additionally, this study also aims to explore cost-effectiveness results of real-world patients treated with pertuzumab in neoadjuvant context.

## Methods

This study included two independent retrospective real-world consecutive cohorts of female patients aged ≥18 years old diagnosed with HER-2 positive BC that received the NeoT between 2012 and 2017 at the Portuguese Oncology Institute of Porto (IPO-Porto) and followed at the institution’s Breast Clinic. IPO-Porto is a national reference public hospital located in the North of Portugal and specialized in the treatment of cancer. The hospital has 11 multidisciplinary specialty units (Porter’s Integrated Practice Unit - IPU), including the Breast Clinic. This clinic started its activities in 2007 and has an independent functional structure with its own areas and specialized staff, allowing patients to receive an integrated and personalized care. In 2018, received 1690 new cases, for a total of approximately 11,000 patients in follow-up, corresponding roughly to 10% of all IPO-Porto patients. Overall, the IPO-Porto BC unit is responsible for treating the greatest number of BC patients in Portugal and is one of the largest BC units of Europe.

**Fig. 1:**
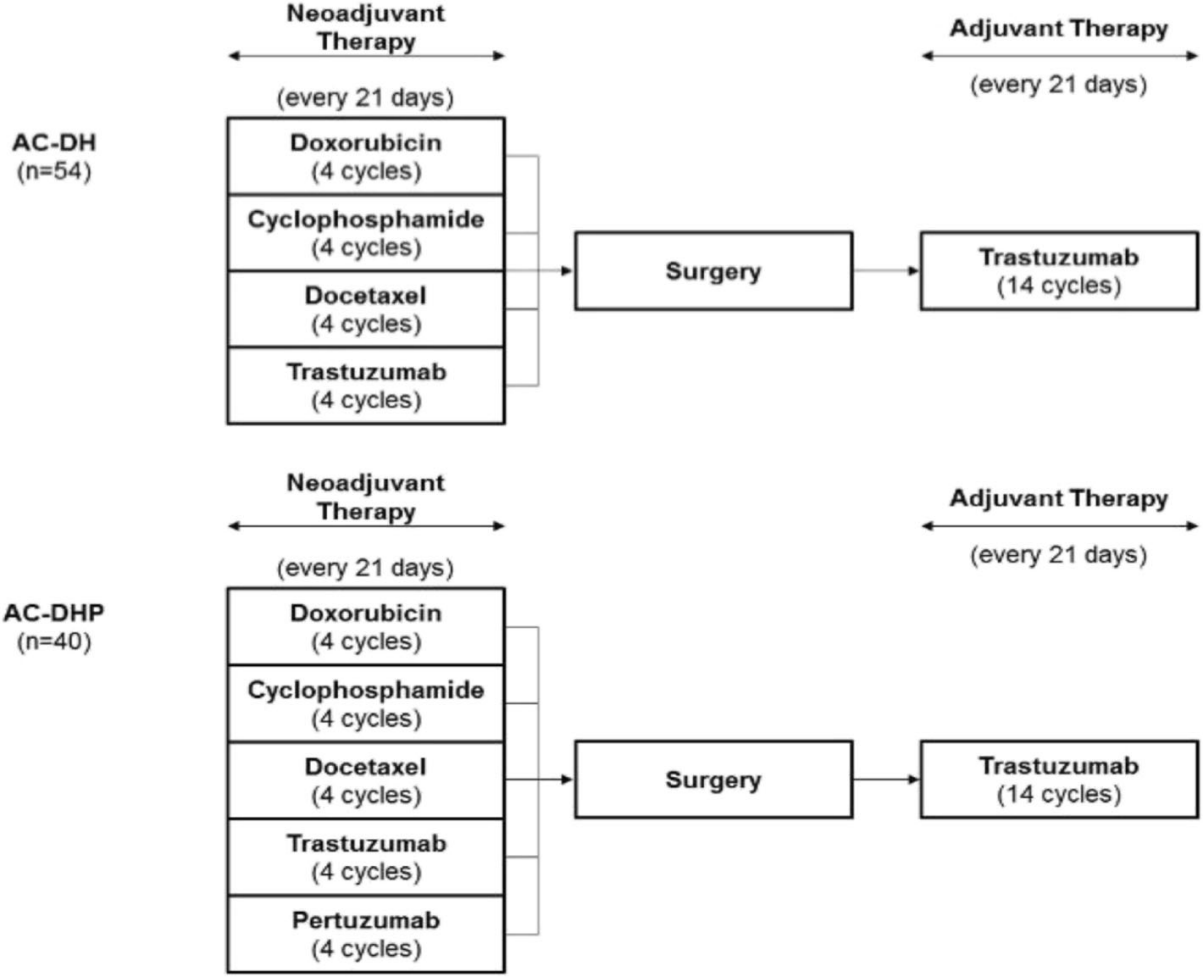
Schematic representation of the pertuzumab treatment protocol. AC-DH: 2012–2015 cohort; A: doxorubicin; C: cyclophosphamide; DH: docetaxel plus trastuzumab; AC-DHP – 2015-2017 cohort; A: doxorubicin; C: cyclophosphamide; DHP - docetaxel, trastuzumab plus pertuzumab

Patients were consecutively included in the study with the diagnosis of operable, locally advanced, or inflammatory stage II or III HER2-positive unilateral BC who performed the complete NeoT at IPO-Porto between January 2012 and mid-2017 were included in the current analysis. All patients received 4 cycles of doxorubicin plus cyclophosphamide followed by 4 cycles of docetaxel. In 2012–2015 period, the regimen included 4 cycles of trastuzumab (AD-DH cohort), while in 2015–2017 (AC-DHP cohort) the patients received 4 cycles of dual HER2-blockade with pertuzumab and trastuzumab as a NeoT in accordance with approved Summary of Product Characteristics [7]. For all patients, the NeoT was followed by surgery and trastuzumab as adjuvant therapy every 21 days, as described in Fig. 1. All patients being treated at the center with one of these protocols between 2012 and 2017 matching inclusion criteria were included in the study.

Exclusion criteria includes patients with stages I or IV at baseline or that performed other chemotherapy treatment rather than above mentioned treatment protocol or that had other cancer diagnosis during study period besides unilateral BC were excluded. Males, clinical trials participants, patients that underwent partial treatment in another institution and non-naïve patients for BC chemotherapy were also excluded. Also, patients with other oncological pathology, and that did not perform the complete pathway at IPO Porto were also excluded. Matching between cohorts was not performed.

This research received approval from the Local Ethics Committee, followed all National ethical standards and procedures, and was performed in accordance with the Helsinki declaration.

### Data collection

Demographic, clinical, treatment and costs data were collected using Excel. The confidentiality and anonymization of data for analysis were ensured.

Data on demographics (age, weight and height), clinical staging/TMN classified by AJCC 7th edition [19], hormone receptors (HR) (positive if estrogen and/or progesterone receptor was > 1%; HER2 was positive if the score was 3+ in immunohistochemistry or in case of 2+ positive by fluorescence in situ hybridization (FISH), Eastern Cooperative Oncology Group (ECOG) Performance Status [20], systemic treatment (date, type of treatment, drug, dose), surgical treatment (mastectomy or conservative, axillary clearance, biopsy of sentinel lymph node, number of positive lymph nodes), and pathological complete response (pCR) as measure of treatment effectiveness were collected. Tumours were classified as operable (T0–3, N-0-1, M0), locally advanced (T2–3, N2–3, M0 or T4a-c, any N, M0) or inflammatory (T4d, any N, M0) following the same criteria from the NeoSphere trial [10].

Cost data information regarding chemotherapy, hormone therapy (includes drugs consumed from outpatient pharmacy and sessions of hospital day), radiotherapy, hospital appointments and exams (includes scheduled consultations, emergency appointments and complementary diagnostic exams), inpatient and outpatient care (includes surgical outpatient care and IPO-Porto homestay costs) were collected to estimate the direct medical costs.

The date of diagnosis was considered as the baseline date and as the starting point for the data collection and medical resource consumption accountability; and the last adjuvant administration of trastuzumab was considered the end of data collection.

### Cost estimation

The micro-costing technique was used, and all medical direct costs from the date of diagnosis to the date of the last administration of adjuvant treatment were accounted individually for each patient.

The unit costs were obtained from government official prices [21–23], except for the drug prices that were obtained from the IPO-Porto costing system - which follows the approved prices by the drug regulator for public hospital drugs (Infarmed). The cost per milligram was calculated and multiplied by the quantity of milligrams administrated per session. All costs were considered for the year of 2017, but hospitalizations/inpatient care and outpatient care episodes were adjusted for this same year using the following formula:

$$ \mathrm{Current}\ \mathrm{price}=\mathrm{Price}\ \mathrm{in}\ \mathrm{legislation}\times \left(1+\mathrm{Variation}\ \mathrm{from}\ \mathrm{current}\ \mathrm{price}\right) $$

By using unit costs, the authors assumed that the unit cost does not differ by patient. For example, the unit cost of a consultation is the same regardless of the duration, even though it may take longer in the beginning of the adjuvant therapy or for older patients. This source of variability was not take into account in the present study.

The outpatient care episodes also include the IPO-Porto homestay costs estimated based on the government official prices.

To determine the total cost per patient, the costs accrued for each variable were summed. The average, median, minimum and maximum cost per patient was obtained for both cohorts. The total cost was calculated by the sum of the costs for each treated patient.

Only medical direct costs from the hospital perspective were considered. Costs were expressed in euros for the year of 2017. The discount rate is not applicable.

### Health outcomes

The clinical outcome measured was the rate of pCR on breast and axilla achieved in last assessment after surgery. The rate was measured by the percentage of patients that achieved the pCR. The pCR is defined as the absence of invasive neoplastic cells or in-situ lesions (ypT0/is ypN0) and is associated with an improved survival, being a suitable outcome for measuring NeoT effectiveness in HER2 positive BC treatment [24, 25].

### Study outcomes

For the main analysis, the average, median, maximum and minimum total cost per patient was calculated for both cohorts. The proportion of the pertuzumab cost in the total costs was calculated by dividing the mean pertuzumab cost by the mean total costs per patient, the mean total chemotherapy costs and the mean of total neoadjuvant costs.

The Incremental Cost-Effectiveness Ratio (ICER) was also estimated by the following formula:

$$ \mathrm{ICER}=\frac{\mathrm{Average}\ {\mathrm{total}\ \mathrm{costs}}_{\mathrm{AC}-\mathrm{DH}\mathrm{P}}-\mathrm{Average}\ {\mathrm{total}\ \mathrm{costs}}_{\mathrm{AC}-\mathrm{DH}}}{\mathrm{pCR}\ {\mathrm{Rate}}_{\mathrm{AC}-\mathrm{DH}\mathrm{P}}-\mathrm{pCR}\ {\mathrm{Rate}}_{\mathrm{AC}-\mathrm{DH}}} $$

### Statistical analysis

Descriptive statistics for categorical variables included frequencies’ tabulation with counts and percentages, and for continuous variables comprised central tendency and dispersion measures. Comparisons between the two cohorts were performed using independent samples t-test for continuous variables. Chi-square or Fisher’s exact tests were used to evaluate the association between two categorical variables.

Binary logistic regression models were used to quantify the relation between patient’s characteristics and pCR. Age, stage of disease at diagnosis, hormone receptors, ECOG, Body Mass Index (BMI), and type of surgery were analysed. Regimen was forced into the optimized model to ensure proper comparison. Exponentiated coefficients (adjusted Odds Ratios (OR)), *p*-value, and 95% confidence intervals (CI) were calculated.

Generalized linear model (GLM) with gamma distribution was used for modelling continuous skewed treatment costs per patient, allowing to test the mean cost increase. All independent variables were included in the model as categorical. The optimized model was obtained by stepwise backward elimination method for selection of independent variables. The adjusted mean cost ratio (MCR), *p*-value, and 95% CI were calculated.

For the binary logistic regression models and for the generalized linear models, first, univariate models were performed and each variable was considered separately. Then, the variables that were considered statistically significant in the univariate models were retained for the multivariable model. The final multivariable model contains the variables that were statistically significant.

A *p*-value < 0.05 was considered statistically significant. Data were computed using STATA V.15 for Windows (StataCorp).

### Sensitivity analysis

The coefficients estimated from multivariable logistic regression were used to perform sensitivity analysis. Sensitivity analysis was performed varying the clinical staging and the HR, obtaining, for each scenario tested, the ICER, the cost per pCR, the number of patients who needed to be treated to have a pCR and the additional cost for pCR. Four scenarios were tested: clinical staging II and negative HR, clinical staging II and positive HR, clinical staging III and negative HR, and clinical staging III and positive HR. The average total direct cost per patient obtained from main analysis was used in all four tested scenarios.

## Results

### Baseline characterization

A total of 40 patients (average age 45.4 years, 67.5% with HR positive, 60.0% with operable and 25.0% with inflammatory tumour) that performed the AC-DHP regimen were included in the analysis. For the AC-DH cohort, a total of 54 patients (average age 50.9 years, 59.3% with HR positive, 27.8% with operable and 24.1% with inflammatory tumour) were included. Baseline characteristics are described in Table 1.

**Table 1:** Patients demographics and clinical characteristics at baseline

	Doxorubicin, cyclophosphamide, docetaxel, trastuzumab plus pertuzumab (*n* = 40)	Doxorubicin, cyclophosphamide, docetaxel plus trastuzumab (*n* = 54)	*P*-value
**Age in years (average ± standard deviation)**	45.4 ± 9.6	50.9 ± 9.9	**0.008**
**Age group (n, %)**			
≤ 24	1 (2.5%)	0 (0.0%)	0.068
25–29	0 (0.0%)	1 (1.9%)	
30–34	2 (5.0%)	1 (1.9%)	
35–39	8 (20.0%)	7 (13.0%)	
40–44	12 (30.0%)	4 (7.4%)	
45–49	5 (12.5%)	12 (22.2%)	
50–54	5 (12.5%)	7 (13.0%)	
55–59	3 (7.5%)	12 (22.2%)	
60–64	2 (5.0%)	4 (7.4%)	
≥ 65	2 (5.0%)	6 (11.1%)	
**BMI (average ± standard deviation)**	25.5 ± 4.7	27.0 ± 5.7	0.168
**ECOG performance status** [20] **(n, %)**			
0	34 (85.0%)	47 (87.0%)	0.777
1	6 (15.0%)	7 (13.0%)	
**Hormonal Receptors (ER and PR) (n, %)**			
At least one positive	27 (67.5%)	32 (59.3%)	0.414
Both negative	13 (32.5%)	22 (40.7%)	
**Clinical staging (n, %)**			
IIA	4 (10.0%)	2 (3.7%)	0.205
IIB	8 (20.0%)	8 (14.8%)	
IIIA	18 (45.0%)	22 (40.7%)	
IIIB	10 (25.0%)	17 (31.5%)	
IIIC	0 (0.0%)	5 (9.3%)	
**Histologic grade (n, %)**			
G1	0 (0.0%)	1 (1.9%)	0.640
G2	11 (27.5%)	19 (35.2%)	
G3	28 (70.0%)	32 (59.2%)	
Undetermined	1 (2.5%)	2 (3.7%)	
**At diagnosis (n, %)**			
Operable	24 (60.0%)	15 (27.8%)	**0.001**
Locally advanced	6 (15.0%)	26 (48.1%)	
Inflammatory	10 (25.0%)	13 (24.1%)	
**Lymph node status (n, %)**			
N0	4 (10.0%)	10 (18.5%)	**0.006**
N1	27 (67.5%)	18 (33.3%)	
N2	9 (22.5%)	21 (38.9%)	
N3	0 (0.0%)	5 (9.3%)	
**Tumour size (n, %)**			
T0	1 (2.5%)	0 (0.0%)	0.420
T1	2 (5.0%)	2 (3.7%)	
T2	13 (32.5%)	11 (20.4%)	
T3	14 (35.0%)	21 (38.9%)	
T4	10 (25.0%)	20 (37.0%)	

BMI: body mass index; ECOG: Eastern Cooperative Oncology Group; ER: estrogen receptor; PR: progesterone receptor. There were no patients with ECOG PS ⩾2

### Health outcomes

The clinical outcomes are described in Table 2. A total of 45.0% of patients treated with AC-DHP regimen presented pCR in breast and axilla, while only 33.3% of patients treated with AC-DH regimen had the same response. Although approximately 35% higher, the pCR difference between both cohorts was not statistically significant for the sample size analysed (*p* > 0.05).

**Table 2:** Clinical outcomes for HER2-positive breast cancer by regimen

	Doxorubicin, cyclophosphamide, docetaxel, trastuzumab plus pertuzumab (*n* = 40)	Doxorubicin, cyclophosphamide, docetaxel plus trastuzumab (*n* = 54)	*P*-value (chi square test)
**Pathological complete response**			
ypT0/is ypN0	18 (45.0%)	18 (33.3%)	0.250
Breast	20 (50.0%)	22 (40.7%)	0.372
Axilla	24 (60.0%)	26 (48.1%)	0.255

Data are number (%). DCIS, ductal carcinoma in situ. nCR, near complete response

pCR separately by breast and axilla were additionally calculated and the results for the pertuzumab cohort were also clinically better in both breast and axilla (pCR in breast: 50% vs. 40.7%; pCR in axilla: 60% vs. 48.1%).

Optimized logistic regression model is presented in Table 3. The treatment regimen was not significantly associated with the pCR in the breast and axilla (ypT0/is ypN0) and pCR in the breast (*p* = 0.304 and *p* = 0.396, respectively), however the association was positive. On the other hand, clinical staging and positive HR were the only patient’s characteristics significantly associated with the achievement of pCR in the breast and axilla (*p* = 0.001 and *p* = 0.003, respectively) and pCR in the breast (p = 0.001 and *p* < 0.001, respectively). The authors opted to present only the variables that were identified as statistically significant predictors and the treatment regimen, which although not significant, was the main variable of interest in this study.

**Table 3:** Optimized Predictive Analysis for pathological complete response and treatment cost per patient

Predictors of pathological complete response (Multivariable logistic regression)				
	**Pathological complete response ypT0/is ypN0**		**Pathological complete response in the breast**	
	**Adjusted OR (95% CI)**	***P*-value**	**Adjusted OR (95% CI)**	**P-value**
**Regimen**				
AC-DH	1		1	
AC-DHP	1.65 (0.63–4.30)	0.304	1.51 (0.58–3.94)	0.396
**Clinical staging**				
II	1		1	
III	0.14 (0.04–0.43)	**0.001**	0.14 (0.04–0.44)	**0.001**
**Hormonal Receptors (ER and PR)**				
Both negative	1		1	
At least one positive	0.21 (0.08–0.59)	**0.003**	0.16 (0.06–0.43)	**< 0.001**
Predictors of treatment cost per patient (Gamma General Model)				
	**Cost per patient**			
**Regimen**	**Adjusted MCR (95% CI)**		**P-value**	
AC-DH	1			
AC-DHP	1.40 (1.30–1.49)		**< 0.001**	

AC-DH, doxorubicin, cyclophosphamide, docetaxel plus trastuzumab. AC-DHP, doxorubicin, cyclophosphamide, docetaxel, trastuzumab plus pertuzumab. ER, estrogen receptor. PR, progesterone receptor

### Study outcomes - costs comparison between cohorts

Mean, median, maximum and minimum total cost per patient for both treatment cohorts are presented in Table 4 and additional graphical representation is presented in Supplementary Fig. 1. Cost comparison was performed between groups and Total cost of session of hospital day, Drug cost for complete regimen and Drug cost for neoadjuvant regimen, associated with Chemotherapy/ Targeted therapy, as well as Conservative treatment cost (Inpatient care), were found to be significantly higher in the AC-DHP cohort. Conversely, Goserelin cost (Hormone Therapy), Total radiotherapy costs and Simple technique cost (Radiotherapy) were higher for the AC-DH cohort. As expected, overall costs were significantly higher in the AC-DHP cohort.

**Table 4:** Costs of HER2-positive breast cancer treatment by regimen

	Doxorubicin, cyclophosphamide, docetaxel plus trastuzumab plus pertuzumab (***n*** = 40)					Doxorubicin, cyclophosphamide, docetaxel, trastuzumab (***n*** = 54)					P-value (average cost per patient)
	Total cost	Minimum cost per patient	Average cost per patient	Median cost per patient	Maximum cost per patient	Total cost	Minimum cost per patient	Average cost per patient	Median cost per patient	Maximum cost per patient	
**Chemotherapy/ Targeted therapy**											
Total cost of session of hospital day	**1,697,525**	**21,501**	**42,438**	**42,187**	**73,656**	**1,515,163**	**9856**	**28,059**	**27,699**	**43,865**	**< 0.001**
Drug cost for complete regimen	1,608,688	20,320	40,217	39,924	71,393	1,394,155	8971	25,818	25,394	41,406	**< 0.001**
Drug cost for neoadjuvant regimen	827,792	13,879	20,695	20,700	27,094	336,884	1654	6239	5846	10,614	**< 0.001**
Doxorubicin cost	1771	34	44	44	53	2446	35	45	45	60	0.266
Cyclophosphamide cost	2585	50	65	64	77	3567	51	66	65	92	0.327
Docetaxel cost	5330	109	133	128	171	7595	93	141	142	193	0.053
Pertuzumab cost	559,124	5855	13,978	14,637	17,564	–	–	–	–	–	
Trastuzumab cost	258,982	4482	6475	6320	9820	323,276	1396	5987	5584	10,274	0.107
Drug cost for adjuvant regimen	780,896	2776	19,522	19,409	48,662	1,057,270	4469	19,949	19,544	35,818	0.691
**Hormone therapy**											
Total cost for hormone therapy treatment^a^	**9857**	**6**	**352**	**28**	**1463**	**10,273**	**14**	**311**	**27**	**1781**	0.780
Anastrozol cost	120	16	20	23	28	460	16	24	23	33	0.086
Exemestane cost	197	64	66	64	69	–	–	–	–	–	
Goserelin cost	4005	320	501	521	641	4966	721	828	801	961	**< 0.001**
Tamoxifen cost	518	21	27	28	35	420	12	26	28	35	0.576
**Radiotherapy**											
Total radiotherapy costs	**200,979**	**3199**	**5153**	**4955**	**8029**	**225,626**	**105**	**4339**	**4098**	**7528**	**0.006**
Complex technique cost	162,094	1004	4156	4015	8029	151,305	1004	3690	3011	7528	0.269
Simple technique cost	38,885	209	1341	1359	2509	74,321	105	1689	1672	2927	**0.041**
**Hospital appointments and Exams**											
Total cost of hospital attendances	**190,415**	**3000**	**4760**	**4848**	**7564**	**242,298**	**2683**	**4487**	**4521**	**6744**	0.175
**Inpatient care**											
Total hospitalizations costs	**106,941**	**1467**	**2674**	**1992**	**7589**	**154,175**	**1007**	**2855**	**1992**	**10,877**	0.617
Hospitalizations cost for breast cancer surgery	81,638	1467	2041	1992	2692	113,054	944	2094	1589	7011	0.801
Mastectomy cost	63,429	1467	2114	1992	2692	99,899	1331	2220	1992	7011	0.679
Conservative treatment cost	18,208	1748	1821	1748	1992	13,155	944	1462	1454	1992	**0.010**
**Outpatient care**											
Total outpatient costs^c^	**49,274**	**289**	**1263**	**289**	**18,544**	**33,710**	**162**	**648**	**289**	**5235**	0.219
**Total cost**	**2,254,991**	**27,487**	**56,375**	**54,819**	**89,543**	**2,181,245**	**15,089**	**40,393**	**40,797**	**60,427**	**< 0.001**

All Values in the table are expressed in Euros - €

^a^Includes drugs consumed from outpatient pharmacy and sessions of hospital day. ^b^Includes scheduled consultations, emergency appointments and complementary diagnostic and treatment exams. ^c^Includes surgical outpatient care and IPO Porto homestay costs

Pertuzumab cost alone was on average 13,978€ per patient, which represents 24.8% of the overall costs per patient (56,375€), 34.8% of the average cost per patient for complete drug regimen (40,217€) and 67.5% of the overall neoadjuvant therapy costs (20,695€). The average cost of all chemotherapy drugs represented the greatest portion of the total costs, reaching 71.3% of the complete treatment costs. The hormone therapy, radiotherapy, appointments, exams, inpatient care, including surgery, and outpatient care were responsible for the 28.7% of the remaining costs. The scenario before pertuzumab followed this trend, with chemotherapy total cost (25,818 €) representing the greatest portion of all treatment costs (40,393€), reaching 63.9%.

The addition of pertuzumab increased in 15,982€ the mean cost per patient, of which 2004€ represented all other medical resources consumed excluding pertuzumab. The adoption of AC-DHP regimen at IPO-Porto increased in 39.6% the overall costs per patient, with 4.7% of the cost increased explained by the other medical resources.

### Sensitivity analysis

From the GLM models we obtained a statistically significant mean cost increase of 40%. Patient’s treatment costs were explained by regimen and, for this reason, sensitivity analysis was conducted only varying the clinical staging and HR. The ICER, as well as sensitivity analysis performed are shown in Table 5. By calculating the ICER, we can conclude that, to increase the pCR rate by one percentage (pp) point, we need to spend on average 1370€.

**Table 5:** Incremental cost-effectiveness ratio (ICER) by regimen and sensitivity analysis

Regimen	Effectiveness	Total direct cost per patient	ICER	Cost per pathological complete response	No. of patients who needed to be treated to have a pathological complete response	Additional cost for pathological complete response
**AC-DH**	33%	40,393 €		121,180 €	3.0	
**AC-DHP**	45%	56,375 €	1370 €	125,277 €	2.2	4097 €
**Sensitivity analysis**						
**Scenario 1. Clinical staging II and negative HR**						
**AC-DH**	85%	40,393 €		47,640 €	1.2	
**AC-DHP**	90%	56,375 €	2955 €	62,502 €	1.1	14,862 €
**Scenario 2. Clinical staging II and positive HR**						
**AC-DH**	54%	40,393 €		74,493 €	1.8	
**AC-DHP**	66%	56,375 €	1338 €	85,205 €	1.5	10,711 €
**Scenario 3. Clinical staging III and negative HR**						
**AC-DH**	43%	40,393 €		93,682 €	2.3	
**AC-DHP**	56%	56,375 €	1282 €	101,427 €	1.8	7745 €
**Scenario 4. Clinical staging III and positive HR**						
**AC-DH**	14%	40,393 €		291,147 €	7.2	
**AC-DHP**	21%	56,375 €	2241 €	268,371 €	4.8	−22,775 €

AC-DH, doxorubicin, cyclophosphamide, docetaxel plus trastuzumab. AC-DHP, doxorubicin, cyclophosphamide, docetaxel, trastuzumab plus pertuzumab. HR, hormonal receptors (include progesterone and estrogen). ICER, incremental cost-effectiveness ratio

Regarding the sensitivity analysis and comparing all the scenarios, lower clinical staging had better results. Moreover, the presence of negative HR is considerably associated with better treatment results when compared to positive HR. Clinical staging II and negative HR had the best effectiveness results for both treatment protocols, but the highest ICER. In this case, to increase the pCR rate by one percentage point, it is necessary to spend on average 2955 €. For the same clinical staging and positive HR, the treatment effectiveness for both cohorts was lower compared to negative HR however the ICER was lower (1338 €).

Regarding clinical staging III, the trend was the same: the presence of positive HR was associated with less treatment effectiveness. However, a more advanced clinical staging inverted the ICER: 1282 € for negative HR and 2241 € for positive HR.

## Discussion

Technological advances in the diagnosis and treatment of BC have not solved the risk of recurrence and high mortality rate of HER2-positive BC. New available treatment options, such as pertuzumab, must be continuously assessed for safety and effectiveness after marketing authorization, which also includes cost-effectiveness. Increasing healthcare costs and resource constraints in the Portuguese NHS [26], lead to debate highly expensive cancer drugs [27]. Assessing the real benefit and price of new drugs is extremely important to the NHS and society as a whole to ensure better resource allocation. Thus, cost-effectiveness and continuous drug performance assessments are important for improving sustainable access to high-value care for patients in need.

Pertuzumab as a NeoT for HER2-positive BC is a relative novelty. Since it is a new treatment, the scarcity of real-world evidence on cost and effectiveness is noticeable. Present results show that patients treated with pertuzumab (AC-DHP protocol) had better clinical outcomes (but non-significant), measured as pCR rates in breast and axilla and pCR rates in breast only, which is in line with the clinical trials results (clinical value) [10, 12]. On the other hand, our study was, to the best of our knowledge, the first to describe a detailed bottom-up analysis of the BC treatment in a public healthcare institution and explore cost-effectiveness of pertuzumab in the real-world setting (economic value).

Accordingly, pertuzumab as NeoT for HER2-positive BC is costly and averages almost one fourth of all treatment costs, one third of chemotherapy and targeted therapy drug costs and more than two thirds of the NeoT costs. Our results are in line with previous research [28] who estimated the real-world treatment cost of metastatic HER2-positive BC from 2004 to 2010 and found that trastuzumab and chemotherapy represented the most significant part of all treatment costs. Pertuzumab was not available in that time frame, but immunotherapy drugs cost weighted heavily in the overall costs. Interestingly, our study identified that the average total cost of chemotherapy and targeted therapy alone in the AC-DHP cohort (42,438 €) was higher than the complete treatment cost in the AC-DH cohort (40,393 €).

The bottom-up approach in this study accurately and comprehensively estimated the institution’s costs with the treatment of HER2-positive BC. Unlike direct costs estimation based on expert opinion or resources consumed in clinical trials, our study relies on a robust and realistic quantification of current treatment costs per patient.

Only direct medical costs are reported here (indirect medical costs were not estimated), which may have led to an underestimation of the overall treatment cost. Data on patient’s follow-up over a longer time horizon was unavailable, since patients in the last cohort (AC-DHP) received NeoT with pertuzumab in 2018. Assessing the impact of BC relapse on costs and survival using RWD was, therefore, not possible.

Nevertheless, the estimated costs are a valuable information for IPO-Porto budgeting and for future HTA assessments in defining drug prices based on its value. In fact, these estimated costs could be used to populate cost-effectiveness models with RWD and accurately estimate simulated long-term horizons with transitions between health states. Our study could ultimately be complemented by indirect costs estimations, additional costs associated with BC relapse and health-related quality of life (HRQOL) data in patients with BC -obtained from current literature- providing better cost-effectiveness and cost-utility treatment assessment.

Concerning the mean healthcare expenditures per patient/year, the estimated costs are impressive versus average healthcare costs in Portugal. The average estimated overall cost per patient of the AC-DHP cohort (56,375€) was 35 times higher than the Portuguese national mean healthcare expenditure per patient/year (1630.05€ in 2016) [29]. Nevertheless, the abovementioned cost underestimation -more evident in a societal perspective- is enough to highlight the high economic burden of BC treatments. This should raise awareness on the importance of technology assessments and resources allocation concerning NHS sustainability.

As for clinical outcomes, pCR results were not statistically significant. This can probably be explained by sample size constraints and baseline differences between cohorts. In addition, the long term benefits of using pertuzumab to reduce BC relapse and increase progression-free survival were not evaluated in this research, despite the important insights on costs.

The sensitivity analysis considered variations in the effectiveness of different sub-groups, changing the clinical staging and positive/negative ER and PR in each tested scenario. Patients with negative HR had better overall clinical results compared with patients with positive HR, which is in line with available literature [30]. The clinical staging II with negative HR resulted in an higher ICER versus clinical staging II with positive HR. This might suggest that adding pertuzumab for the treatment of clinical staging II with negative HR may not bring significant cost-effectiveness benefits when compared with positive HR cases in the same clinical staging. However, we must consider that long-term scenarios can generate better results for pertuzumab considering the reduction of receding and increasing PFS.

Conversely, the results in clinical staging III patients showed a higher ICER for positive HR cases, although the effectiveness was considerably different in favour of patients with HR negative in the clinical staging III. Pertuzumab may not be as advantageous for the treatment of clinical staging III patients with positive HR, as in the case of negative HR patients, but broader samples are needed. Broader samples size would also allow for results with greater statistical impact. Nonetheless, this limitation could not be avoided since the sample considered represented all eligible patients from IPO-Porto in the selected period. Since this is based on RWE data, stratification and baseline adjustment of characteristics were limited. As treatment protocols evolve over time and become more complex, based on available evidence, patient profile changes. Baseline differences between both cohorts limited some clinical comparisons. Protocol evolution can also explain a 5.7% cost increase when not accounting for pertuzumab, despite costs adjustment to the same year.

Additionally, although IPO-Porto is a reference center for cancer treatment, present results depict the clinical practice of a single institution, which may not be a full representation of the Portuguese context. Using specific IPO-Porto reference prices can be seen as a limitation, but since used for both treatment cohorts, it has no impact in the results.

Despite these limitations, higher clinical effectiveness of AC-DHP cohort in all scenarios and optimized model results with controlled baseline characteristics indicate clinically meaningful but non-significant results in favour of pertuzumab. However, this also stresses the need for more RWE for pertuzumab as NeoT. These results are a stepping stone in the RWE monitoring of cost-effectiveness for pertuzumab as a NeoT. Future research in collaboration with other national and international institutions should aim for more robust results by increasing sample size and the observation time. In the context of breast cancer, larger time horizons are best suited for exploring different outcomes, such as overall survival and impacts beyond healthcare, in the economic and social context of patients. IPO-Porto mission is aligned with current VBHC trends and patient centricity is at the core of the therapeutic approach. Future research should also collect Patient Reported Outcome Measures (such as HRQOL), which are vital to better decision-making in healthcare resource allocation.

Public oncology hospitals, like IPO-Porto, play a relevant societal role by providing specialized care for a devastating disease and by encouraging policy making and payment based on patient outcomes. This is an ongoing process done in collaboration with different stakeholders for better decision making, so patients can ultimately benefit from innovative but cost-effective therapies.

Neoadjuvant pertuzumab indication was approved in Europe in 2015 and, from an economic perspective, it received different recommendations from reference HTA bodies. The National Institute for Health and Care Excellence (NICE) in the UK [31] positively recommended the use of pertuzumab for NeoT, but received a negative recommendation from The National Centre for Pharmacoeconomics (NCPE) in Ireland [32]. Portuguese INFARMED is yet to publish the HTA appraisal for pertuzumab in NeoT.

## Conclusion

Our study is important for the monitoring of pertuzumab treatment costs and its performance in the real-world context, considering the growing concern with the NHS sustainability. Although this new technology represents a significant investment, and safety and effectiveness should be continuously monitored, the availability of this treatment is very positive for clinical outcomes. Incorporating innovative therapies is essential for the quality of the universal health coverage in Portugal. Additionally, our study gave insights of the cost-effectiveness of adding neoadjuvant pertuzumab in clinical practice. Future research using pertuzumab as comparator for newly treatments in the HTA and reimbursement assessment based on patient’s outcomes in Portugal will surely consider the present results.

## Supplementary Information

**Additional file 1: Supplemental Fig. 1.** Treatment cost with/without pertuzumab and average cost per patient. AC-DH, adriamycin, cyclophosphamide, docetaxel plus trastuzumab. AC-DHP, adriamycin, cyclophosphamide, docetaxel, trastuzumab plus pertuzumab.

## Data Availability

The datasets used and/or analysed during the current study are available from the corresponding author on reasonable request.

## Abbreviations

ACT: Adjuvant chemotherapy
BC: Breast cancer
CI: Confidence intervals
ECOG: Eastern Cooperative Oncology Group
ER: Estrogen receptor
FISH: Fluorescence in situ hybridization
GLM: Generalized linear model
HER2: Human epidermal growth factor receptor 2
HR: Hormonal receptors
HRQOL: Health-related quality of life
HTA: Health Technology Assessment
ICER: Incremental Cost-Effectiveness Ratio
nCR: Near complete response
NCPE: The National Centre for Pharmacoeconomics
NeoT: Neoadjuvant treatment
NHS: National Health System
NICE: The National Institute for Health and Care Excellence
OR: Adjusted Odds Ratios
OS: Overall survival
pCR: Pathological complete response
PFS: Progression-free survival
RWD: Real world data
VBHC: Value-Based Healthcare
