# Supplementary material for: The addition of neoadjuvant pertuzumab for the treatment of HER2+ breast cancer: a cost estimate with real-world data
Source: Health Econ Rev. 2021 Sep 10;11:33. doi: 10.1186/s13561-021-00332-0 (PMC8431932; doi:10.1186/s13561-021-00332-0)

mental figure 1. Treatment cost with/without pertuzumab and average cost per patient

AC-DH, adriamycin, cyclophosphamide, docetaxel plus trastuzumab. AC-DHP, adriamycin, cyclophosphamide, docetaxel, trastuzumab plus pertuzumab.


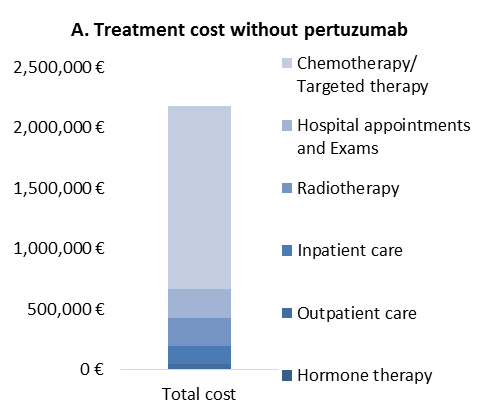

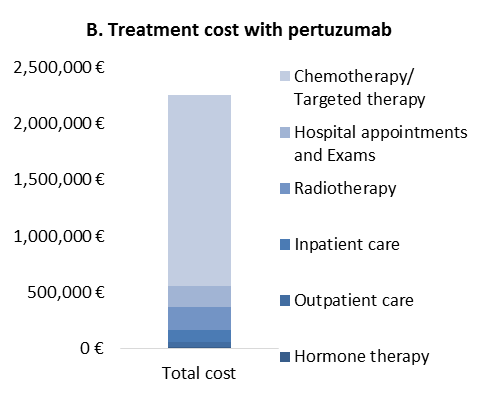

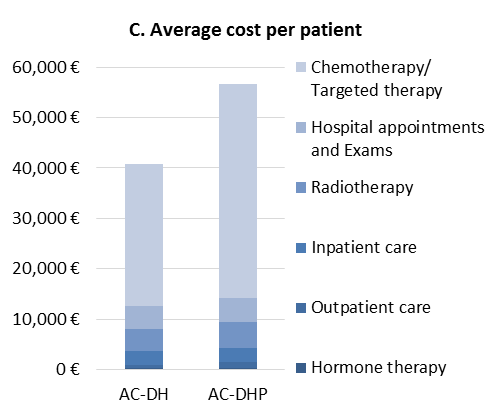

Supplement: Supplementary file 1 — Additional file 1: Supplemental Fig. 1. Treatment cost with/without pertuzumab and average cost per patient. AC-DH, adriamycin, cyclophosphamide, docetaxel plus trastuzumab. AC-DHP, adriamycin, cyclophosphamide, docetaxel, trastuzumab plus pertuzumab. [file 13561_2021_332_MOESM1_ESM.docx]
